# Practical ultrasonographic technique to precisely identify and differentiate tendons and ligaments of the elbow at the level of the humeral epicondyles: anatomical study

**DOI:** 10.1007/s00256-020-03693-5

**Published:** 2020-12-11

**Authors:** Patrick Omoumi, Pedro Augusto Gondim Teixeira, Samuel R. Ward, Debbie Trudell, Donald Resnick

**Affiliations:** 1 Department of Diagnostic and Interventional Radiology, Lausanne University Hospital and University of Lausanne, Rue du Bugnon 46, 1011 Lausanne, Switzerland; 2 Service d’imagerie Guilloz, CHU Hôpital Central, 29, avenue du Maréchal De Lattre De Tassigny, 54035 Nancy, France; 3 Departments of Orthopaedic Surgery and Radiology, UC San Diego, 9500 Gilman Drive, La Jolla, CA 92093-0863 USA; 4 Department of Radiology, Teleradiology / HCOP - University of California, 408 Dickinson Street, San Diego, CA 92103 USA

**Keywords:** Ultrasound, Elbow, Tendon, Ligament, Anatomy, Epicondylitis

## Abstract

**Objectives:**

To develop a practical step-by-step technique to precisely identify and differentiate tendons and ligaments attaching to the humeral epicondyles, to confirm through gross anatomical study the accurate structure identification provided by this technique and to determine the frequency at which each structure can be identified in healthy volunteers.

**Materials and methods:**

First, ten fresh frozen cadavers (6 men, age at death = 58–92 years) were examined by two musculoskeletal radiologists and a step-by-step technique for the identification of tendons and ligaments at the level of humeral epicondyles was developed. Second, the accurate identification of structures was confirmed through gross anatomical study including anatomical sections on five specimens and layer-by-layer dissection technique on five others. Finally, 12 healthy volunteers (6 men, average age = 36, range = 28–52) were scanned by two radiologists following the same technique.

**Results:**

An ultrasonographic technique based on the recognition of bony landmarks and the use of ultrasonographic signs to differentiate overlapping structures was developed and validated through gross anatomical study. In healthy volunteers, most tendons and ligaments were identified and well-defined in ≥ 80% of cases, except for the extensor carpi radialis brevis and extensor digiti minimi tendons on the lateral epicondyle (having common attachments with the extensor digitorum communis) and the palmaris longus tendon on the medial epicondyle (absent, or common attachment with the flexor carpi radialis).

**Conclusion:**

A step-by-step approach to the ultrasonographic assessment of tendons and ligaments at the humeral epicondyles allowed accurate identification of and differentiation among these structures, in particular those relevant to pathological conditions.

## Introduction

The anatomy of the tissues about the medial and lateral epicondyles of the elbow is complex, consisting of an intricate assembly of numerous tendons and ligaments confined to a relatively small area. The common extensor tendon and lateral collateral ligamentous complex attach to the lateral epicondyle, while the common flexor tendon and medial collateral ligamentous complex attach to the medial epicondyle. While tendons and collateral ligaments are intimate at their attachment, the precise extension of a lesion may have implications in patient management and prognosis [1–4]. Lateral epicondylitis and medial epicondylitis are terms that are used to describe the commonly encountered alterations that affect the adjacent extensor and flexor tendons, respectively, and the accompanying imaging findings are especially helpful in atypical cases or when symptoms are refractory to conservative treatment [5]. The role of imaging is then to confirm the diagnosis, exclude other conditions, and potentially guide therapeutic injections [5]. Moreover, accurate localization of the pathologic processes that affect these tissues may be important in the presurgical assessment, decreasing the risk of treatment failure [1, 6].

Despite its potential clinical impact, the precise identification of specific tendons and ligaments adjacent to the humeral epicondyles is challenging in practice, regardless of the imaging modality [7].

Magnetic resonance imaging (MRI) and ultrasonography are the modalities of choice for the assessment of the tendons and ligaments about the medial and lateral epicondyles [8–13]. While some authors have shown that the attachment sites of these tendons and ligaments could be facilitated by the use of bony landmarks at MRI, to the best of our knowledge, such investigation has not been performed using ultrasonography [14, 15]. In theory, ultrasonography benefits from various advantages compared to MRI, including its higher spatial resolution and the ability to readily obtain views that are specific to the long and short axes of each tendon and ligament [10]. The bony landmarks previously described at MRI to facilitate structure differentiation should also be visible at ultrasonography. Furthermore, the anisotropy artifact that affects tendons and ligaments at ultrasonography may be useful for structure differentiation: overlapping structures with slightly different orientations should have different echogenicity [16]. Finally, the interfaces between overlapping structures might be echogenic on ultrasonography, due to interposing fatty connective tissue that may surround tendons and ligaments [17]. Consequently, we hypothesized that the identification of tendons and ligaments close to their epicondylar insertions would be possible using ultrasonography.

Therefore, our aim was (1) to develop a practical step-by-step technique to identify ligaments and tendons attaching to the humeral epicondyles, based on (a) the recognition of previously published bony landmarks (to differentiate structures in the anteroposterior/craniocaudal axes), and (b) the use of echogenic lines and difference in echogenicity (to differentiate overlapping structures); (2) to confirm through gross anatomical study the accurate structure identification provided by this step-by-step ultrasonographic technique; and (3) to determine the frequency at which each structure can be identified in healthy volunteers.

## Materials and methods

The first part of this study was performed on donated cadavers, derived from persons about whom there was little information, excepts for age and gender, and therefore did not require ethical committee approval at the institution where it was carried out. The study on healthy volunteers was approved by our institutional ethical committee with informed consent obtained from participants.

### Specimen preparation

Ten human elbow joint specimens were obtained from seven fresh frozen cadavers (six men and one woman; age at death: 58–92 years). The specimens consisted of the wrist, forearm, elbow joint, and distal half of the upper arm and were deep-frozen at − 40 °C for at least 3 days (Forma Bio-Freezer; Forma Scientific, Marietta, Ohio). All specimens were allowed to thaw for 24 h at room temperature before ultrasonography. Immediately after imaging, the specimens were frozen again at − 40 °C for at least 3 days.

### Ultrasonography

Ultrasonography was performed using an IU22 system (Philips, Best, Netherlands) and a high frequency transducer (17–5 MHz), wrapped in a latex pouch. The examination was performed at a frequency of 17 MHz.

Two musculoskeletal radiologists (initials anonymized for review), with at least 2 years of experienced in musculoskeletal ultrasonography at the time of the study, performed the examinations and evaluated the images in consensus. All structures were examined in both the long- and short-axis planes. The elbows were examined in a semi-flexed position.

**Fig. 1:**
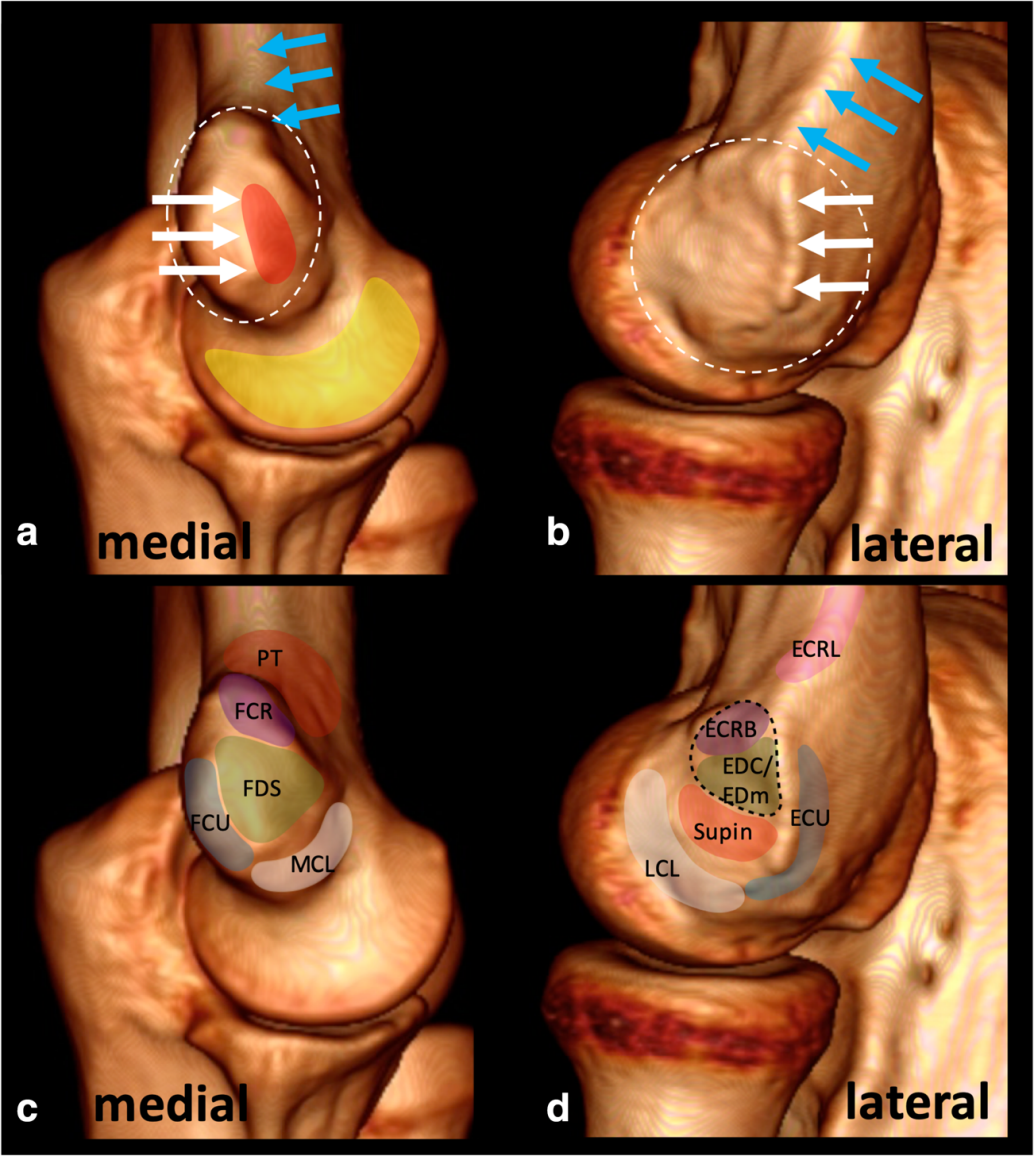
Anatomy of the medial (a) and lateral (b) epicondyles and the respective tendon and ligament attachment sites (c and d) shown on a CT of the elbow with surface rendering. a and b show the bony landmarks that are consistently visible at ultrasonography: the medial and lateral epicondyles (dotted circles), the supracondylar ridge (blue arrows), the epicondylar ridge (white arrows), the anterolateral flat area (red) and the semilunar area (yellow). The distribution of tendon and ligament attachments on the medial and lateral epicondyles is indicated on c and b. The ECRB and the EDC/EDm tendons have a conjoint tendon (dashed line in d). PT: pronator teres, FCR: flexor carpi radialis, FDS: flexor digitorum superficialis, PL: palmaris longus, FCU: flexor carpi ulnaris, MCL: medial collateral ligamentous complex, ECRL: extensor carpi radialis longus, ECRB: extensor carpi radialis brevis, EDC: extensor digitorum communis, EDm: extensor digiti minimi, Supin: supinator tendon, ECU: extensor carpi ulnaris, LCL: lateral collateral ligamentous complex

The extensor and flexor muscles of the arm were located at the wrist and followed proximally towards their insertions in the humeral epicondyles. The examiners then sought to differentiate these tendons and the collateral ligamentous complexes using three sets of signs: specific osseous landmarks in the medial and lateral epicondyles, hyperechogenic or hypoechogenic lines, and differences in echogenicity. The definition of the osseous landmarks of the epicondyles was based on previously published literature and included for the lateral epicondyle: the supracondylar ridge, the epicondylar ridge, and the anterior, superior, and posterior tubercles, and for the medial epicondyle: the supracondylar ridge, the epicondylar ridge, the anterosuperior and anteroinferior tubercles, the semilunar area, and the flat area [14, 15]. The attachment sites of tendons and ligaments relative to the bony landmarks, as previously described in the literature, were assessed at ultrasonography (Fig. 1) [14, 15]. Echogenic lines were defined as hyperechogenic or hypoechogenic lines separating the structures attaching to the epicondyles. Difference in the echogenicity of these structures was based on the presence of anisotropy artifact related to slightly different orientations of these structures and enhanced by slight angulation of the probe.

### Gross anatomical study

Five specimens were sectioned with a band saw into 3-mm-thick slices in the coronal plane with fluoroscopic guidance. Photographs and radiographs (Faxitron HP 43805 N X-Ray System, Hewlett-Packard, Palo Alto CA, USA; tube current, 30 kV; exposition time, 30 s) of all sections were obtained.

Five other specimens were dissected by an anatomist (anonymized). The progressive layer-by-layer dissection technique described by De Maeseneer et al. was used [18]. All structures were scanned by ultrasonography before and after they were individually removed to verify their correct identification by ultrasonography. The same ultrasonography device and probe that were used in the scanning of the whole specimens were used for the staged dissection.

### Healthy volunteers

Twelve elbow joints from 12 healthy volunteers (6 men, average age 36, range 28–52) were examined by the two radiologists in consensus, following the scanning technique developed in the first part of the study, and using the same ultrasonography device and probe. The identification of each structure on the four views on each humeral condyle was graded from 0 to 2 as follows: 0 = not visible, 1 = visible but poorly defined, and 2: visible and well-defined. The visibility of each tendon was considered good if the average score was ≥ 1.5.

## Results

### Cadaveric study: ultrasonography

#### Lateral epicondyle

**Fig. 2:**
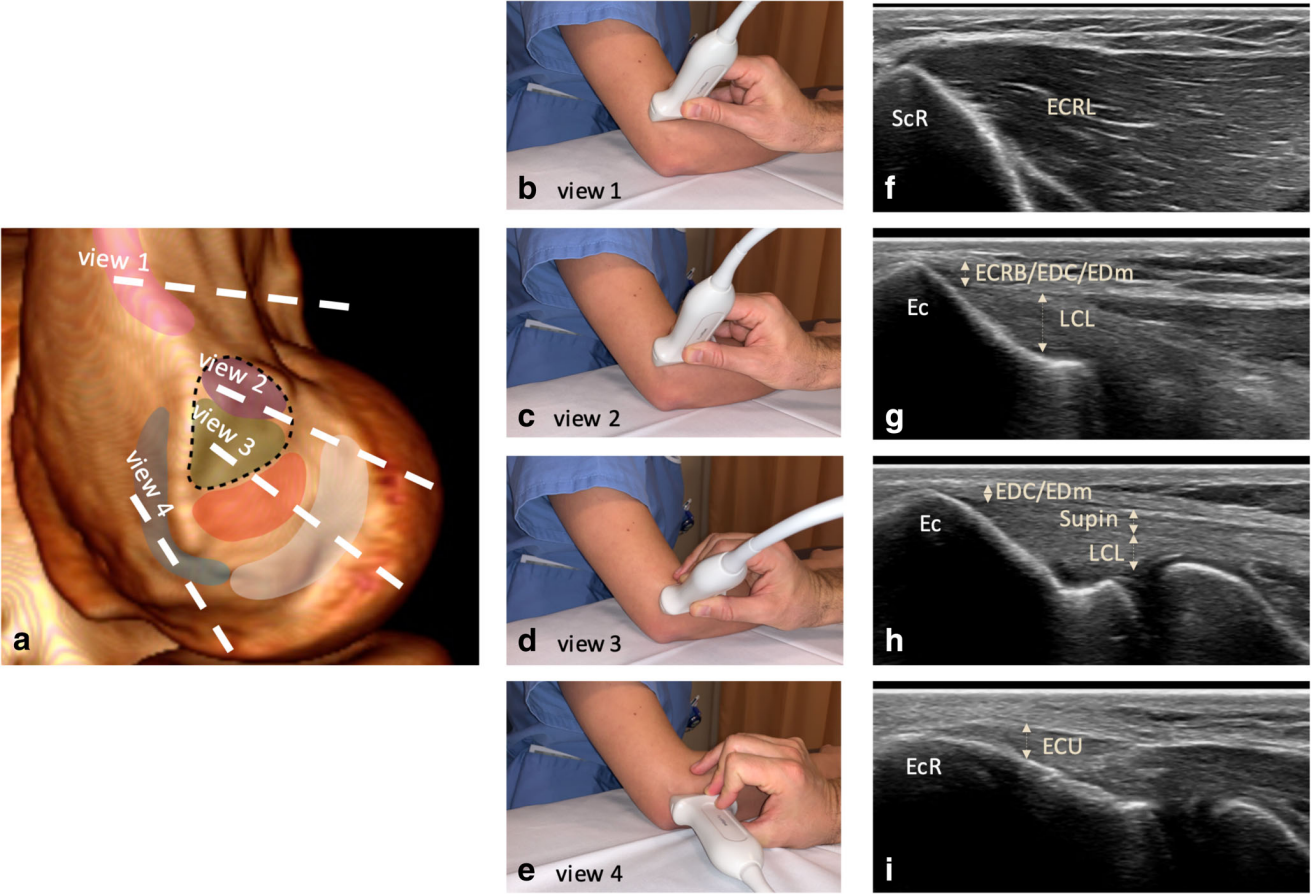
Ultrasonographic assessment technique for the lateral epicondyle with position of the different ultrasonographic views on the lateral epicondyle shown on a CT with surface rendering (**a**), on photographs of a volunteer (**b**–**e**), with corresponding ultrasonographic images (**f**–**i**): View 1: supracondylar ridge (easily identified in the short-axis plane of the distal humerus): the muscular attachment of the ECRL onto the supracondylar ridge is identified. View 2: anterosuperior aspect of epicondyle (slightly moving the probe distally to the epicondyle from View 1, at its anterior aspect): the conjoint ECRB/EDC/EDm tendon is visible. View 3: intermediate view (between views 2 and 4): from superficial to deep, the EDC/EDm tendons, supinator tendon and LCL are visible. View 4: posterior aspect of epicondylar ridge: From surface to deep, the ECU tendon can be easily identified. ECRL: Extensor carpi radialis longus, ECRB: extensor carpi radialis brevis, EDC: extensor digitorum communis, EDm: extensor digiti minimi, Supin: supinator tendon, ECU: extensor carpi ulnaris, LCL: lateral collateral ligamentous complex, ScR : supracondylar ridge, Ec : epicondyle, EcR : epicondylar ridge

Following is a detailed description of ultrasonographic assessment of the lateral epicondyle, which led to the development of the step-by-step technique described in Fig. 2.

The following bony landmarks that had been previously described in the literature were identified by ultrasonography: supracondylar ridge (identified in 100% of our 10 specimens), epicondylar ridge (100%), superior tubercle (40%), posterior tubercle (50%), anterior tubercle (visible in 5 out of 8 specimens). The presence of intratendinous calcifications precluded the visualization of the anterior tubercle in two specimens.

The supracondylar ridge was easily identified in the short axis plane of the humerus. Two muscles were found inserting on the supracondylar ridge: the more proximal brachioradialis muscle and the more distal extensor carpi radialis longus (ECRL) muscle. The ECRL muscle had no identifiable tendon, and, because of this feature, could be differentiated from the other extensor tendons without difficulty.

The attachment of the extensor carpi radialis brevis (ECRB) tendon was found at the most superior aspect of the epicondylar ridge, including the region of the anterior tubercle. The ECRB tendon was conjoint with the extensor digitorum communis (EDC) and extensor digiti minimi (EDm) tendons, from which it could not be separated close to the insertion site. Further away from the insertion, the EDC and EDm tendons had a slightly more oblique course, and the bulk of their insertion was slightly more inferior compared to that of the ECRB tendon.

The insertion of the extensor carpi ulnaris (ECU) tendon was located at the posteroinferior aspect of the lateral epicondyle, posterior to the epicondylar ridge.

The lateral, or radial, collateral ligamentous complex (LCL) had a broad insertion at the inferior aspect of the epicondyle, and located underneath the extensor tendons. It could be differentiated from the overlying extensor tendons in 90% of the cases based on the presence of a hyperechogenic line between the two structures and/or a difference in the echogenicity. In the one specimen in which these structures could not be differentiated, a 13-mm calcification in the common extensor tendon was present, which precluded the visualization of the underlying ligament.

The insertion of the supinator tendon in the lateral epicondyle was located between the insertions of the EDC/EDm tendon and that of the LCL, and differences in echogenicity allowed the accurate differentiation of these structures.

#### Medial epicondyle

**Fig. 3:**
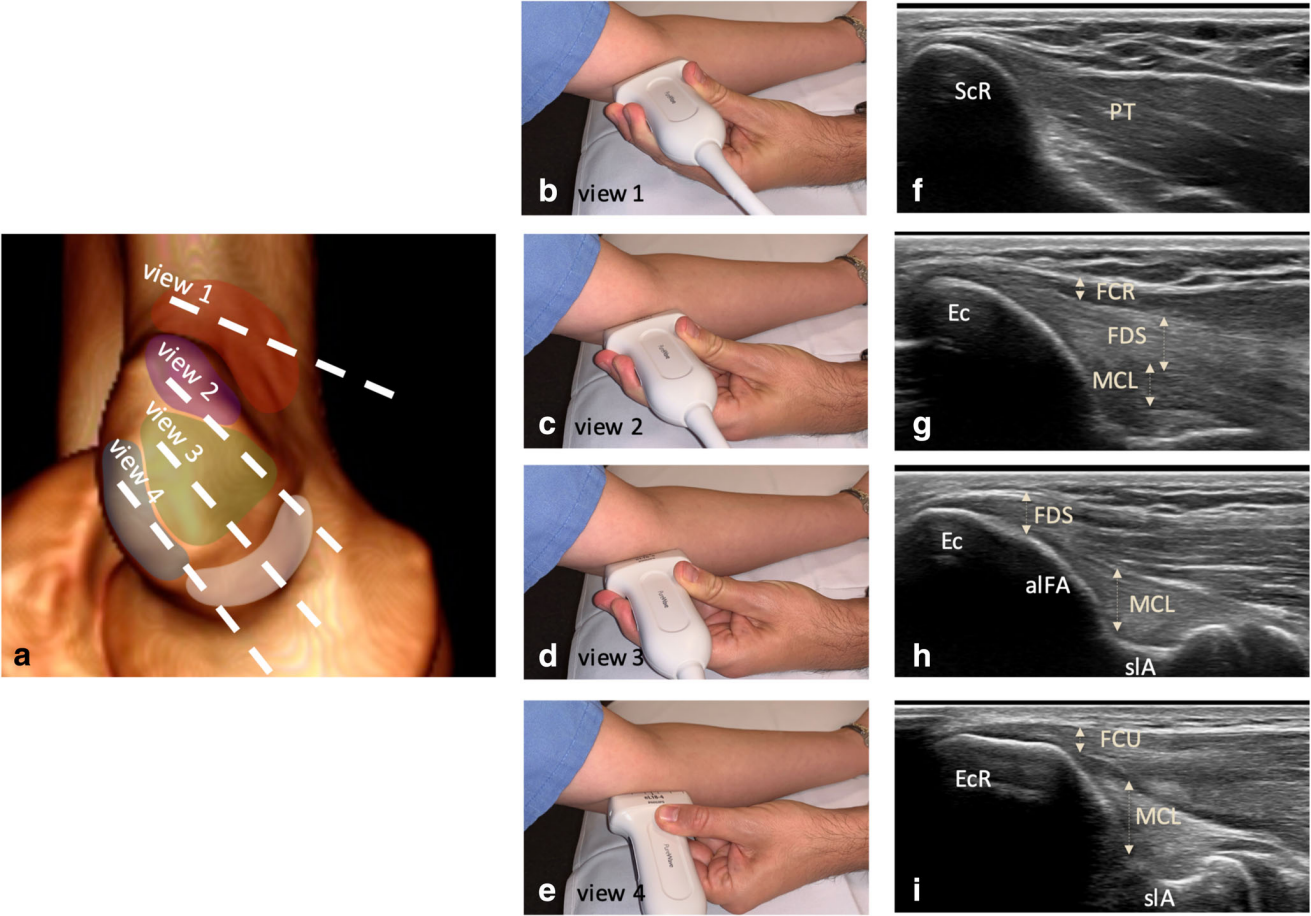
Ultrasonographic assessment technique for the medial epicondyle with position of the different ultrasonographic views on the lateral epicondyle shown on a CT with surface rendering (**a**), on photographs of a volunteer (**b**–**e**), with corresponding ultrasonographic images (**f**–**i**): View 1: supracondylar ridge (easily identified in the short-axis plane of the distal humerus): the muscular attachment of the pronator teres onto the supracondylar ridge is identified. View 2: anterosuperior aspect of epicondyle (slightly moving the probe distally to the epicondyle from View 1, at its anterior aspect): from superficial to deep, three structures can be differentiated by the presence of echogenic lines/difference of echogenicity: the FCR tendon, the FDS tendon and the MCL. View 3: intermediate view (between views 2 and 4): from superficial to deep, three structures can be differentiated by the presence of echogenic lines/difference of echogenicity: the palmaris longus tendon (inconsistent; not visible here), the FDS tendon and the MCL. View 4: epicondylar ridge: from surface to deep, the FCU tendon and the MCL can be easily identified. FCR: flexor carpi radialis, FDS: flexor digitorum superficialis, PL: palmaris longus, MCL: medial collateral ligamentous complex, FCU: flexor carpi ulnaris, ScR : supracondylar ridge, Ec : epicondyle, EcR : epicondylar ridge

Following is a detailed description of ultrasonographic assessment of the medial epicondyle, which led to the development of the step-by-step technique described in Fig. 3.

The following bony landmarks, which have been previously described in the literature, were identified by ultrasonography: supracondylar ridge (identified in 100% of our 10 specimens), epicondylar ridge (100%), anterolateral flat area (100%), semilunar area (100%), and anterosuperior (60%) and anteroinferior (80%) tubercles.

The supracondylar ridge was easily identified in all specimens in the short-axis/transverse plane of the humerus. Anterior to it and superior to the anterosuperior tubercle when present, the mainly muscular attachment of the pronator teres (PT) was easily identified in the long-axis plane of the muscle. Below the supracondylar ridge, the epicondylar ridge was easily identified in the short-axis plane of the humerus in all specimens. The flexor carpi radialis (FCR) tendon was located in the most anterior part of the epicondylar ridge, and the bulk of its fibers attached to the anterosuperior tubercle, when present. The palmaris longus (PL) tendon has been reported to have a common attachment with the FCR tendon and was not easily identified in these specimens [14]. The flexor digitorum superficialis (FDS) tendon attached on the epicondylar ridge, caudal to the attachment of the FCR tendon, in the anteroinferior tubercular area when present. The insertion site of the flexor carpi ulnaris (FCU) tendon was located at the posterior aspect of the epicondylar ridge.

The medial, or ulnar, collateral ligamentous complex (MCL) attached inferiorly to a broad region in the semilunar area, and it was identified by its obliquely oriented surface in the inferior portion of the medial epicondyle. The MCL could be separated from the flexor tendons in all specimens. This differentiation was made by both the presence of an overlying hyperechogenic line and a difference in the echogenicity of the MCL relative to the adjacent tendons. The anterior bundle of the MCL was readily visualized in its entirety, extending from its attachment in the anterior part of the semilunar area to its insertion in the sublime tubercle and ulnar ridge. In most specimens, this bundle could be separated from the joint capsule by a hyperechogenic line. The posterior bundle of the MCL was more difficult to assess with ultrasonography. It was better evaluated through a posterior approach, as a hypoechogenic band forming the floor of the cubital tunnel.

### Cadaveric study: gross anatomical study

**Fig. 4:**
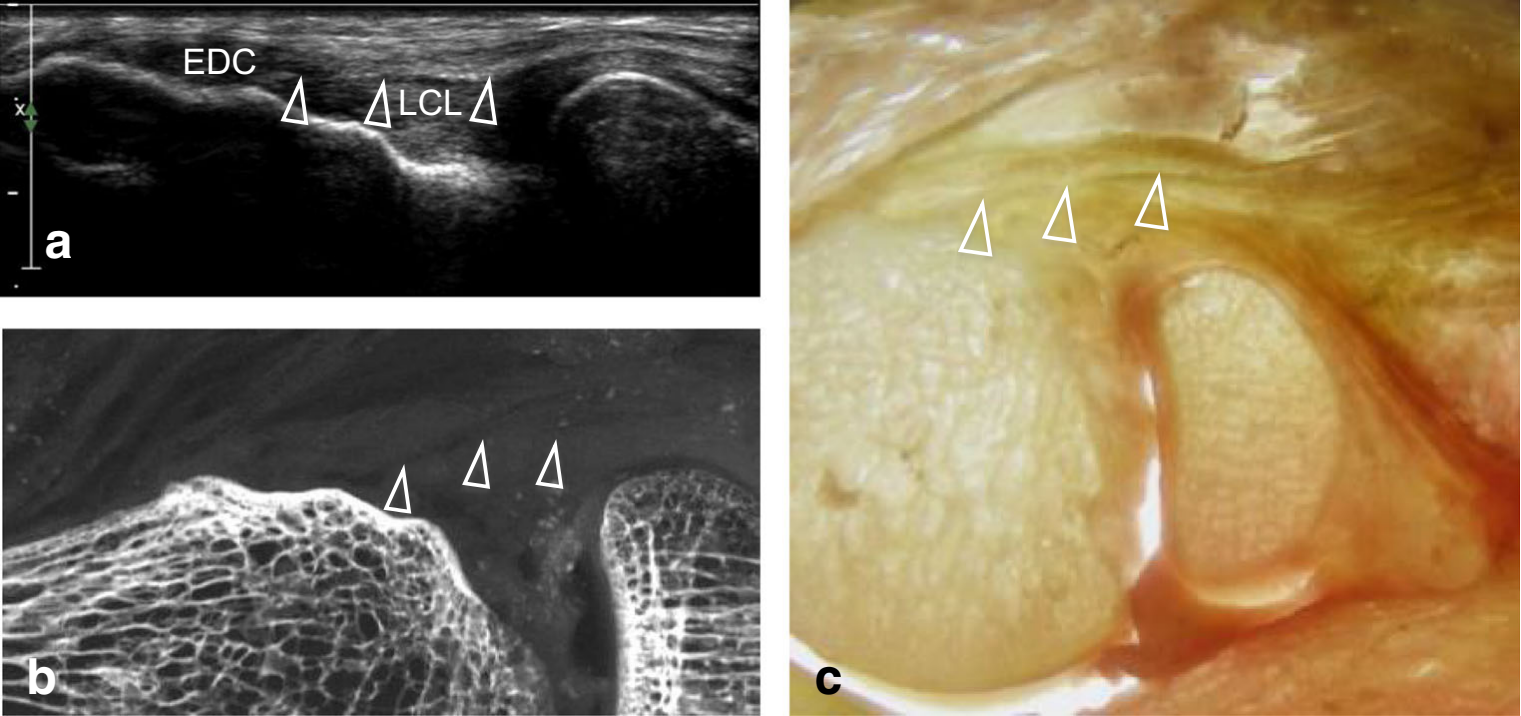
Identification of a hyperechogenic line between the lateral collateral ligamentous complex (LCL) and the overlying extensor digitorum communis (EDC) tendon at ultrasonography (**a**) corresponding to a radiolucent line on radiograph (**b**), likely corresponding to a fat layer (arrowheads). The interface is also seen on the backlit gross anatomical slice (**c**)

These gross anatomical studies allowed us to confirm the anatomy of the tendons, ligaments, and their respective attachments based on osseous landmarks that have been previously described in the literature and, further, the accurate identification of these structures and attachments by ultrasonography [14, 15] (Fig. 4).

**Fig. 5:**
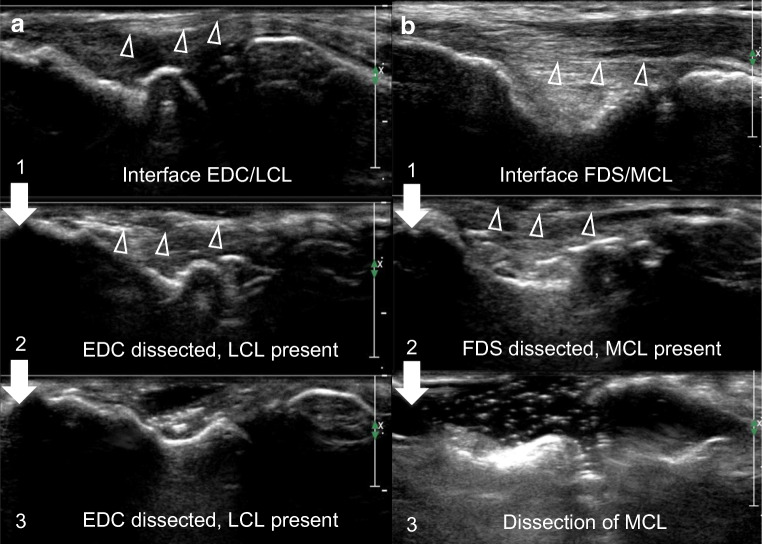
Progressive layer by layer dissection technique. A progressive layer by layer dissection technique is presented for the lateral (**a**) and medial (**b**) epicondyles. Each structure is identified at ultrasonography. The identified structure is then resected at dissection. The ultrasonography is repeated, with the confirmation of the correct identification of anatomical structures. In **a**, arrowheads point at the interface between the EDC and LCL. In **b**, arrows point at the interface between the FDS and the MCL. EDC: extensor digitorum communis, LCL: lateral collateral ligamentous complex, FDS: flexor digitorum superficialis, MCL: medial collateral ligamentous complex

The progressive layer-by-layer dissection technique allowed us to confirm the differentiation of superficial and deep structures by the difference of echogenicity and/or presence of hyper/hypo echogenic lines between these structures, as described in Fig. 5.

### Healthy volunteers

Detailed results of the identification of each structure on each of the four views are reported in Tables 1 and 2. On both the lateral and medial epicondyle, most structures were identified and well-defined on at least one of the views in 80% of cases or more (except for the ECRB and EDm tendons on the lateral epicondyle (identified in 50 and 30% of cases, respectively) and the PL tendon on the medial epicondyle (identified in 50% of cases).

**Table 1:** Frequency and grade of visibility of the tendinous/ligamentous insertions on the lateral epicondyle on each ultrasonographic view (data are percentage of visibility in 12 elbows from healthy volunteers, with visibility grade in parenthesis).

	ECRL	ECRB	EDC	EDm	Supin	ECU	LCL
Not Visible (grade 0)		50%		70%			
Visible on View 1	100% (2)						
Visible on View 2		50% (1.4)	40% (2)		90% (1.3)		90% (1.1)
Visible on View 3			80% (1.9)	30% (2)	30% (2)		100% (1.9)
Visible on View 4						100% (1.8)	90% (1.4)

*ECRL* Extensor carpi radialis longus, *ECRB* extensor carpi radialis brevis, *EDC* extensor digitorum communis, *EDm* extensor digiti minimi, *Supin* supinator tendon, *ECU* Extensor carpi ulnaris, *LCL* lateral collateral ligamentous complex

**Table 2:** Frequency and grade of visibility of the tendinous/ligamentous insertions on the medial epicondyle on each ultrasonographic view (data are percentage of visibility in 12 elbows from healthy volunteers, with visibility grade in parenthesis).

	PT	FCR	FDS	PL	FCU	MCL
Not Visible (grade 0)				50%		
Visible on View 1	100% (2)					
Visible on View 2		90% (2)		10% (2)		80% (1.25)
Visible on View 3		10% (2)	90% (1.89)	50% (2)		100% (1.6)
Visible on View 4			30% (1.5)		100% (1.8)	100% (1.4)

*PT* pronator teres, *FCR* flexor carpi radialis, *FDS* flexor digitorum superficialis, *PL* palmaris longus, *FCU* flexor carpi ulnaris, *MCL* medial collateral ligamentous complex

## Discussion

In this study, we describe a step-by-step ultrasonographic method for the differentiation of tendons and ligaments about the medial and lateral epicondyles of the humerus. This method is based on the recognition of bony landmarks consistently present in cadavers and patients, to which tendons and ligaments attach with a known distribution in the craniocaudal and anteroposterior axes. These landmarks serve to identify the expected location of tendon and ligament attachments. To further differentiate overlapping structures in different views, echogenic lines and differences in echogenicity were used. Applied to volunteers, this technique allowed us to differentiate the structures that are relevant to pathological conditions such as the LCL and MCL in 100% of cases, the ECRB/EDC tendons in up to 80% of case, and the PT muscle and FCR tendinous attachments in 90% of cases.

It should be noted that the ECRB and EDm tendons were properly visualized in 50% and 30% of volunteers, respectively. This was due to the fact that the ECRB and EDm tendons had a conjoint tendon with the EDC tendon, as shown in the cadavers, which is in line with previous anatomical studies [15, 19]. On the medial side, the palmaris longus was identified in 50% of volunteers. This was due in some cases to the absence of this tendon as a normal variant, or in other cases to the fact that the palmaris tendon had a conjoint insertion with the FCR tendon, in keeping with previous reports [14].

The correct differentiation among the structures inserting in the humeral epicondyles can help to accurately determine the extension of pathology in these structures. This information may be of clinical significance, as has been emphasized in the orthopedic literature [1, 6, 8, 19]. For instance, the tendons most frequently affected by pathological processes about the epicondyles are well known. In lateral epicondylitis (or tennis elbow), the ECRB tendon is almost invariably affected, whereas the EDC tendon is affected in 35% of the cases [6, 19]. In medial epicondylitis (or golfer’s elbow), most of the changes are localized in the PT or FCR musculotendinous attachments [8]. Furthermore, some cases of treatment failure in lateral epicondylitis are thought to be related to the lack of a complete excision of pathological tissue at the time of surgery, increasing the significance of accurately localizing the involved structures [6]. Ultrasonography is indicated to confirm the diagnosis in refractory or doubtful cases of lateral epicondylitis. It also has been used to guide intratendinous therapeutic injections and to monitor therapy. A better understanding of the sonographic anatomy of these structures and their differentiation may, therefore, be useful for the management of epicondylitis.

To our knowledge, there are no reports of the performance of MRI in differentiating the structures about the lateral and medial epicondyles, with the exception of two papers focusing on the use of osseous landmarks [14, 15]. In these reports, however, a clear differentiation among all structures was not possible, probably due to the limited resolution of MRI relative to the size of the structures being assessed. Despite the higher resolution of ultrasonography, previously published studies on elbow ultrasonography did not seek to differentiate among the components of the common extensor and flexor tendons, tendons whose individual components are known to be difficult to separate [7]. There are a few reports that confirm success in the differentiation of the LCL and MCL from the overlying tendons but, to our knowledge, no clear criteria were defined for that differentiation [10, 20–22].

A classical technique for structure identification with ultrasonography is to follow structures from a location where they can easily be recognized to the region of interest [10]. This technique, however, is difficult to apply to the common extensor and flexor tendons owing to the great number of structures that merge to attach on a small surface area in the corresponding epicondyle. These difficulties served as our rationale to utilize ultrasonography and the knowledge of the attachment sites in the craniocaudal and anteroposterior axes, as well as the differentiation of overlapping structures with use of echogenic lines and differences in echogenicity. The value of echogenic lines in the differentiation between overlapping tendons or between tendons and underlying ligaments has not been studied previously. These lines may be caused to the presence of fatty connective tissue at the interface between overlapping structures, as seen in some of our specimens. As to the difference in echogenicity between some of these structures, it is likely related to the difference in their fiber orientation as previously described, creating anisotropy artifacts that can be used to differentiate between them [10].

Furthermore, it should be mentioned that while our technique focused on the assessment of these anatomical structures in their long axis, the ultrasonographic technique should always include an examination in their short axis, as with any other structures.

This study had some limitations. First, because of the small number of specimens and healthy volunteers, our sample is not representative of the general population, even though our findings correlated with those in previous reports. Further studies are necessary to fully assess the differentiation of these structures in a larger cohort of subjects. Second, we did not assess persons with abnormalities of the flexor or extensor tendons or medial or lateral ligaments, in which the echogenicity of individual structures may vary. Third, we did not attempt to differentiate the components of the LCL (the lateral ulnar collateral ligament, and the radial collateral ligament) which attach on the epicondyles. The ultrasonographic assessment of the LCL and its components has been previously reported [13].

In conclusion, we describe a step-by-step approach to the ultrasonographic assessment of tendons and ligaments about the humeral epicondyles, with an emphasis on accurate identification of and differentiation among these structures. The approach is based on the combination of previous knowledge from MRI regarding the osseous landmarks and specific sonographic signs including difference in echogenicity and the presence echogenic lines at interfaces between structures. Knowledge of the distribution of tendon and ligament attachments along the epicondyles, and of their anatomical relationships helps to more accurately determine the precise localization of a variety of pathological processes that affect these tendons and ligaments and that are often present in persons presenting with medial or lateral epicondylitis. Such localization should allow more appropriate therapeutic intervention, including ultrasonography-guided interventions.

## References

[1] Fowler KAB, Chung CB (2006). Normal MR imaging anatomy of the elbow. Radiol Clin N Am.

[2] Bredella MA, Tirman PF, Fritz RC, Feller JF, Wischer TK, Genant HK (1999). MR imaging findings of lateral ulnar collateral ligament abnormalities in patients with lateral epicondylitis. AJR Am J Roentgenol.

[3] Bunata RE, Brown DS, Capelo R (2007). Anatomic factors related to the cause of tennis elbow. J Bone Joint Surg Am.

[4] Cohen MS, Romeo AA, Hennigan SP, Gordon M (2008). Lateral epicondylitis: anatomic relationships of the extensor tendon origins and implications for arthroscopic treatment. J Shoulder Elb Surg.

[5] Taylor SA, Hannafin JA (2012). Evaluation and management of elbow tendinopathy. Sports Health.

[6] Organ SW, Nirschl RP, Kraushaar BS, Guidi EJ (1997). Salvage surgery for lateral tennis elbow. Am J Sports Med.

[7] Bianchi S, Martinoli C, Baert AL (2007). Ultrasound of the musculoskeletal system.

[8] Park G-Y, Lee S-M, Lee MY (2008). Diagnostic value of ultrasonography for clinical medial epicondylitis. Arch Phys Med Rehabil.

[9] Tran N, Chow K (2007). Ultrasonography of the elbow. Semin Musculoskelet Radiol.

[10] Connell D, Burke F, Coombes P (2001). Sonographic examination of lateral epicondylitis. AJR Am J Roentgenol.

[11] Jacobson JA, Propeck T, Jamadar DA, Jebson PJL, Hayes CW (2003). US of the anterior bundle of the ulnar collateral ligament: findings in five cadaver elbows with MR arthrographic and anatomic comparison--initial observations. Radiology..

[12] Miller TT, Adler RS, Friedman L (2004). Sonography of injury of the ulnar collateral ligament of the elbow-initial experience. Skelet Radiol.

[13] Gondim Teixeira PA, Omoumi P, Trudell DJ, et al. Ultrasound assessment of the lateral collateral ligamentous complex of the elbow: imaging aspects in cadavers and normal volunteers. Eur Radiol. 2011. 10.1007/s00330-011-2076-8 PMC3101344 21318472

[14] Buck FM, Zoner CS, Cardoso F (2010). Can osseous landmarks in the distal medial humerus be used to identify the attachment sites of ligaments and tendons: paleopathologic-anatomic imaging study in cadavers. Skelet Radiol..

[15] Zoner CS, Buck FM, Cardoso FN (2010). Detailed MRI-anatomic study of the lateral epicondyle of the elbow and its tendinous and ligamentous attachments in cadavers. AJR Am J Roentgenol.

[16] Lin J, Fessell DP, Jacobson JA, Weadock WJ, Hayes CW (2000). An illustrated tutorial of musculoskeletal sonography: part I, introduction and general principles. AJR Am J Roentgenol.

[17] Hodgson RJ, O’Connor PJ, Grainger AJ (2012). Tendon and ligament imaging. Br J Radiol.

[18] De Maeseneer M, Jager T, Vanderdood K, Van Roy P, Shahabpour M, Marcelis S (2004). Ultrasound during dissection of cadaveric specimens: a new method for obtaining ultrasound-anatomic correlations in musculoskeletal radiology. Eur Radiol.

[19] Greenbaum B, Itamura J, Vangsness CT, Tibone J, Atkinson R (1999). Extensor carpi radialis brevis. An anatomical analysis of its origin. J Bone Joint Surg (Br).

[20] Miller TT, Shapiro MA, Schultz E, Kalish PE (2002). Comparison of sonography and MRI for diagnosing epicondylitis. J Clin Ultrasound.

[21] Martinoli C, Bianchi S, Giovagnorio F, Pugliese F (2001). Ultrasound of the elbow. Skelet Radiol.

[22] Finlay K, Ferri M, Friedman L (2004). Ultrasound of the elbow. Skelet Radiol.

